# Human stem cell-based retina on chip as new translational model for validation of AAV retinal gene therapy vectors

**DOI:** 10.1016/j.stemcr.2021.08.008

**Published:** 2021-09-14

**Authors:** Kevin Achberger, Madalena Cipriano, Matthias J. Düchs, Christian Schön, Stefan Michelfelder, Birgit Stierstorfer, Thorsten Lamla, Stefan G. Kauschke, Johanna Chuchuy, Julia Roosz, Lena Mesch, Virginia Cora, Selin Pars, Natalia Pashkovskaia, Serena Corti, Sophia-Marie Hartmann, Alexander Kleger, Sebastian Kreuz, Udo Maier, Stefan Liebau, Peter Loskill

**Affiliations:** 1 Institute of Neuroanatomy & Developmental Biology (INDB), Eberhard Karls University Tübingen, Tübingen, Germany; 2 Department of Biomedical Engineering, Faculty of Medicine, Eberhard Karls University Tübingen, Tübingen, Germany; 3 Boehringer Ingelheim Pharma GmbH & Co. KG, Biberach an der Riss, Germany; 4 NMI Natural and Medical Sciences Institute at the University of Tübingen, Reutlingen, Germany; 5 Department of Internal Medicine I, University Hospital Ulm, Ulm, Germany; 6 3R-Center for In vitro Models and Alternatives to Animal Testing, Eberhard Karls University Tübingen, Tübingen, Germany

**Keywords:** retina on chip, human iPSC, retinal organoids, AAV vectors, gene therapy, organ-on-chip

## Abstract

Gene therapies using adeno-associated viruses (AAVs) are among the most promising strategies to treat or even cure hereditary and acquired retinal diseases. However, the development of new efficient AAV vectors is slow and costly, largely because of the lack of suitable non-clinical models. By faithfully recreating structure and function of human tissues, human induced pluripotent stem cell (iPSC)-derived retinal organoids could become an essential part of the test cascade addressing translational aspects. Organ-on-chip (OoC) technology further provides the capability to recapitulate microphysiological tissue environments as well as a precise control over structural and temporal parameters. By employing our recently developed retina on chip that merges organoid and OoC technology, we analyzed the efficacy, kinetics, and cell tropism of seven first- and second-generation AAV vectors. The presented data demonstrate the potential of iPSC-based OoC models as the next generation of screening platforms for future gene therapeutic studies.

## Highlights

• The retina on chip (RoC) combines iPSC-organoid and organ-on-chip technology
• We characterized seven adeno-associated viruses (AAVs) in terms of transduction efficacy and cell tropism
• The RoC is a novel translational tool for AAV vector characterization
• The presented workflow could be a blueprint for future drug development

## Teaser

Loskill and colleagues demonstrate that the retina on chip combining hiPSC-organoid and organ-on-chip technologies can serve as a novel translational tool for characterization and validation of AAV vectors by mimicking clinically relevant application routes. Seven AAVs were characterized in terms of transduction efficacy, cell tropism, and pharmacokinetics, showcasing a workflow that could serve as blueprint for future drug and gene therapy development.

## Introduction

Retinal diseases are the most common cause of visual impairment in developed countries and have become the worldwide leading cause of childhood blindness (Gilbert and Foster, 2001). Beside significant economic costs, these conditions have enormous consequences on the quality of life for the affected patients. Especially the absence of effective therapies resulting in poor prognosis creates a high emotional burden.

Therefore, renewed efforts to find treatment options are constantly being undertaken. Despite being in the early stages, molecular diagnosis and new treatment strategies such as optogenetics, cell transplants, and gene therapy have already shown the first promising results. In 2017, the first gene therapy for Leber congenital amaurosis was approved by the US Food and Drug Administration (FDA) (Trapani and Auricchio, 2018).

Considered an easily accessible, highly compartmentalized and immune-privileged organ, the eye is a particularly promising target for gene therapy. Adeno-associated virus (AAV) vectors have become the gold standard for gene therapy addressing eye diseases, largely due to their favorable safety profiles, superior transduction capacity, and long-lived gene expression (Trapani and Auricchio, 2018). To date, the majority of clinical trials deliver AAV vectors via intravitreal or subretinal application (Ochakovski et al., 2017). Subretinal applications lead to a localized and highly efficient transduction of photoreceptors and the retinal pigment epithelium (RPE) and are therefore used in most clinical trials addressing genetic disorders affecting these cell types. However, this complex procedure comes with a risk for complications due to retinal detachment during the operation (Garafalo et al., 2020). Conversely, the intravitreal application, which is routinely used for application of therapeutics, has the potential for a much broader biodistribution of applied vectors and is considered as relatively safe. However, intravitreal injection with naturally occurring serotypes shows overall poor transduction efficacy for retinal cells (Lipinski et al., 2013), resulting in low expression of therapeutic proteins, and this limits the potential for successful gene therapies. Capsid-modified, next-generation AAV vectors generated by screening of complex capsid libraries (Büning and Srivastava, 2019; Michelfelder and Trepel, 2009) are a promising approach to overcome the lack of efficacy. However, translation of results generated in preclinical models to the patient is still a major challenge (Dalkara et al., 2013; Hordeaux et al., 2018). So far, the field has almost exclusively relied on mice as preclinical screening models to identify novel AAV vectors. Two promising approaches to overcome the lack of translatability are (1) switching to non-human primates (NHPs) closer resembling the human physiology (Byrne et al., 2020), and (2) utilizing human-relevant *in vitro* models based on human (induced pluripotent stem cell [iPSC]-derived) retinal cells.

Self-assembling stratified organ-like tissues termed organoids derived from iPSCs have brought a new level of complexity to *in vitro* studies. Particularly retinal organoids (ROs) constitute a prime example of the vast possibilities offered by the organoid technology. They contain all known major retinal cell types, including photoreceptor cells, bipolar cells, Müller glia, and ganglion cells, and possess an *in vivo*-like retinal layering (Zhong et al., 2014). Most importantly, ROs build functional synaptic connections and are photosensitive. Recent studies assessing human fetal retina tissues and ROs side by side demonstrated the organoid's close resemblance of retinal development and maturation to an advanced embryonic stage (Cowan et al., 2020).

The advantage of accurate *in vitro* manipulation and the human origin renders ROs very suitable for assessing efficacy, cell toxicity, and cell tropism in non-clinical models of gene therapy. However, cultivation in suspension in dish culture has major limitations. The poor media-to-tissue ratio, lack of vasculature, and the unavoidable media change do not allow the study of long-term effects of a given drug or gene therapeutics.

Besides relying on developmental biology to generate microphysiological tissues, microfabrication engineering provides a more controlled approach. By integrating human tissues into tailored microfluidic platforms, organ-on-chip (OoC) technologies recapitulate physiological tissue structure and function as well as vasculature perfusion. In the past decade, a variety of OoCs mimicking different types of tissues, organ functions, and pathologies have been introduced (Marx et al., 2020; Zhang et al., 2018). Due to their human relevance, amenability for experimental studies, and high-content characteristic, they provide an immense potential for future drug development (Franzen et al., 2019; Low et al., 2020), especially in the field of ophthalmology (Haderspeck et al., 2019).

However, one of the current limitations of OoC technology is the difficulty to generate complex stratified 3D tissues featuring a number of different cell types. This challenge can be addressed by incorporating organoids into OoC platforms. The integration of organoids into a controlled micro-environment of OoCs featuring vasculature-like perfusion and *in vivo* transport processes also solves major challenges of the organoid technology. By addressing the limitations of both technologies, the synergistic combination of OoCs and organoids paves the way for the next generation of stem cell-based microphysiological systems (Achberger et al., 2019; Takebe et al., 2017).

Here, we demonstrate for the first time that complex human iPSC-based OoCs can be utilized to test transduction efficacy of gene therapy in a pharmaceutical setting. Therefore, we employed a human retina-on-chip (RoC) model integrating iPSC-ROs and RPE cells in a tailored microfluidic platform to test the performance of different types of AAV vectors. The compartmentalization and vasculature-like perfusion of the system enabled a physiological subretinal-like injection of the AAV particles and a nutrient supply via a choroidal-like vasculature. The optical accessibility provided the opportunity for *in situ* live-cell imaging. Additionally, we present results from initial screening steps on human iPSC (hiPSC)-ROs providing higher throughput with lower complexity, a workflow that could serve as a blueprint for drug development pipelines in future.

## Results

### AAV2, AAV2.7m8, and ShH10 show expression and cellular tropism after intravitreal application in mouse retina

**Figure 1:**
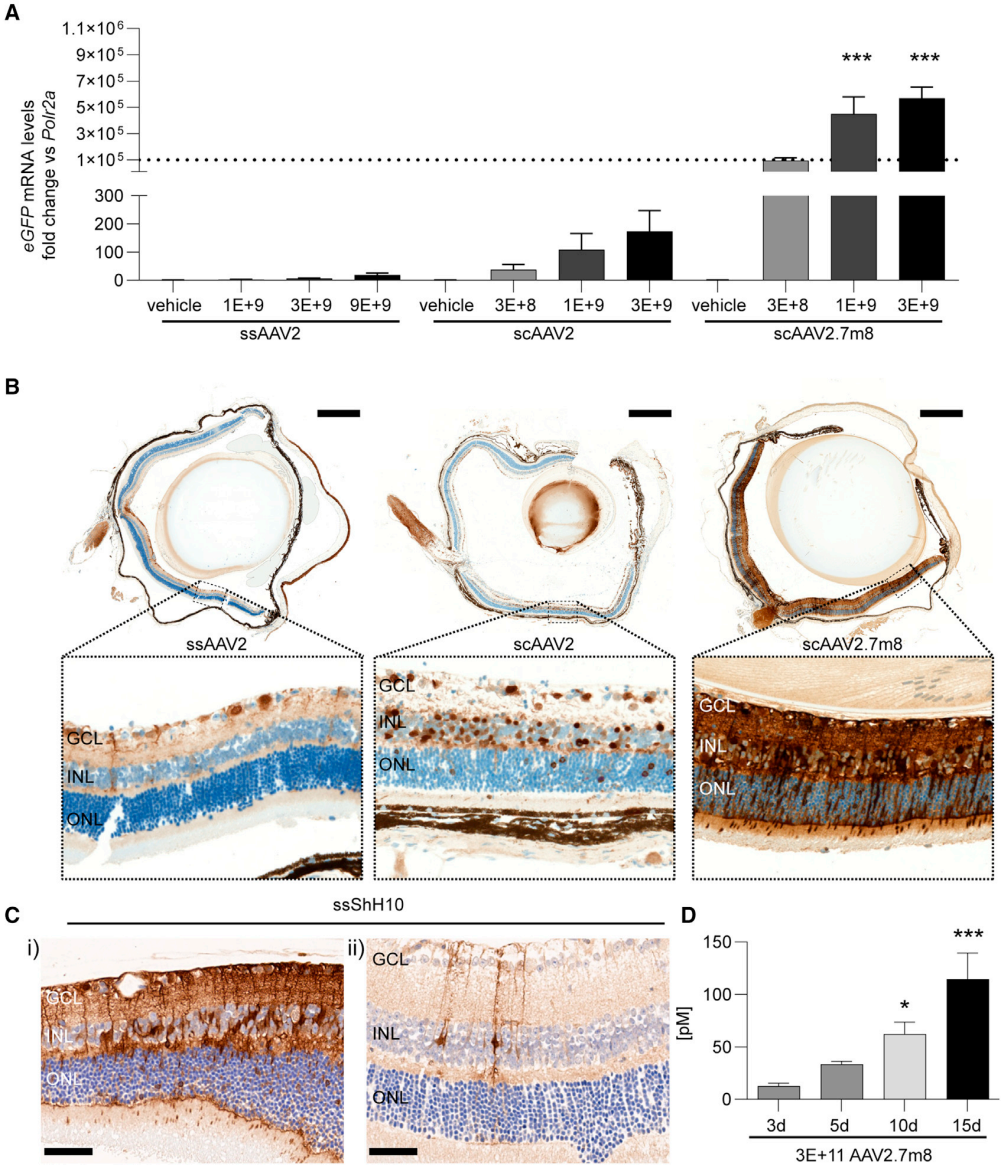
AAV-induced expression in the mouse eye after intravitreal application (A) *eGFP* RNA levels 3 weeks after injection. Statistical analysis in the graph represents the comparison with scAAV2 at similar doses. (B) Representative vertical sections of mouse eyes 3 weeks after injection of 3 × 10^9^ vector genomes. Section were stained with anti-eGFP antibody (DAB, brown) and hematoxylin for cell nuclei (blue). Scale bars: (top) 500 μm, (bottom) 50 μm. (C) Histological staining for eGFP in the mouse retina after (i) 5 × 10^9^ vector genomes and (ii) 1 × 10^9^ vector genomes of ssShH10. Scale bars: 50 μm. (D) Expression of secreted anti-FITC antibody over time course of 15 days. Statistical analysis represents comparison with day 3. (A, C) n = 5–6 eyes from five to six animals per condition, mean + SEM.

First, performance of generated recombinant AAV vectors was analyzed in the eyes of adult mice. Therefore, different doses of AAV2 harboring either linear single-stranded AAV2 (ssAAV2) or self-complementary (sc) AAV2 (scAAV2) and AAV2.7m8 with self-complementary sequence of eGFP (scAAV2.7m8) under the control of the cytomegalovirus (CMV) promotor were subjected to intravitreal application (Figure 1). Three weeks after vector application, analyses of mRNA levels and histological stainings of the eye showed that all vectors expressed eGFP in a dose-dependent manner (Figure 1A). Comparison of similar vector doses showed that switching from a linear single-stranded (ss) eGFP sequence to an sc sequence led to a marked increase in expression. Significantly higher eGFP levels were detected when sequences were delivered with the scAAV2.7m8 versus the scAAV2 capsid (Figures 1A and 1B). Of particular interest is the cell-type-specific expression, which can enable additional therapeutic approaches. ShH10 was described in rat models as preferentially transducing Müller glia (MüG) after intravitreal application (Klimczak et al., 2009). In this study, histological analysis of mouse retinas for eGFP expression confirmed a strong cellular tropism of ss ShH10 (ssShH10) vectors for MüG cells (Figure 1C). Transduction of MüG was observed applying a high (Figure 1C, i) and low dose (Figure 1C, ii) of ssShH10.

The constant increase of expression in the eye over the course of 15 days was verified by expression analysis of anti-fluorescein isothiocyanate (FITC) antibody protein in eye homogenate (Figure 1D). The sequence for the anti-FITC antibody was packed in an scAAV2.7m8 under the control of the CMV promotor. Comparison of days 3, 10, and 15 showed a highly significant increase of anti-FITC antibody protein.

### AAV2, ShH10, AAV2.7m8, but not AAV9 efficiently transduce human ROs *in vitro*

Initially, before moving into the RoC, we performed first-line experiments in dish-cultured ROs. For this proof-of-concept investigation, four AAV serotypes containing an sc CMV-eGFP expression cassette were selected: scAAV2, scAAV9, scShH10, and scAAV2.7m8.

As ROs show different cellular compositions depending on their age, experiments were performed in ROs with two defined developmental stages: day 80 ROs containing retinal progenitors, immature PRC, amacrine cells, and ganglion cells, and day 300 ROs containing all retinal cell types with no or only rare observations of ganglion cells (Cowan et al., 2020).

**Figure 2:**
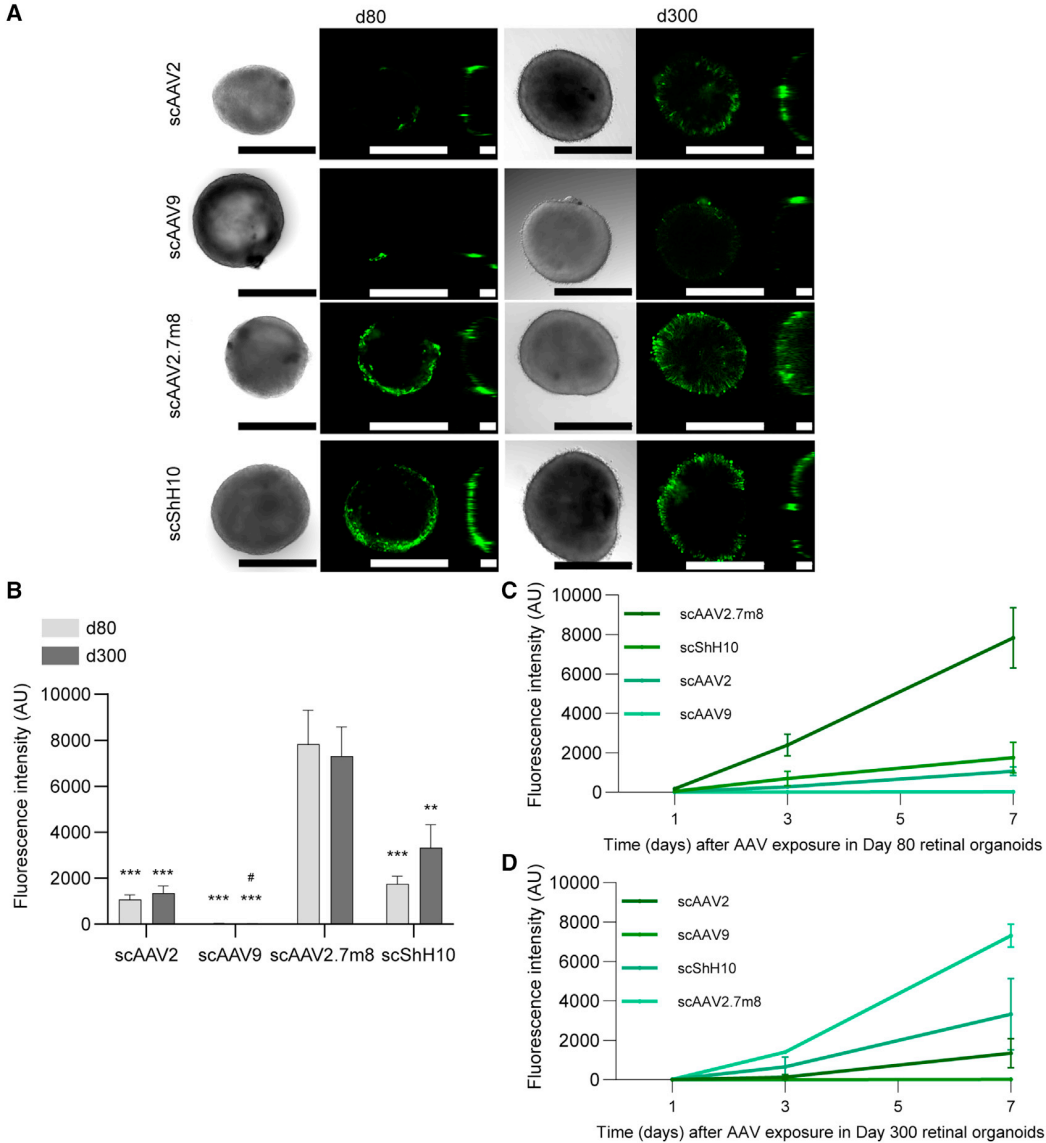
eGFP expression in AAV-treated ROs differentiated for 80 and 300 days (A) Confocal imaging of AAV-treated organoids expressing eGFP (green). Left, brightfield; middle, x-y projection; right, y-z projection. Scale bars: 500 μm. (B–D) (B) Quantification of mean eGFP fluorescence in day 80 and day 300 ROs exposed for 1, 2, or 3 days to the respective AAV (1 × 10^10^ virus genomes/well) and quantified after 7 days. Statistics represent the comparison between AAV for the same RO age with scAAV2.7m8 (^∗^) and with scShh10 (#). Kinetics of eGFP for (C) day 80 ROs and (D) day 300 ROs. (B–D) n = 17–18 separate ROs per condition, mean + SEM. See also Figures S2 and S3.

ROs were imaged by brightfield and confocal high-resolution microscopy to assess general morphology and overall eGFP expression pattern (Figure 2A). The confocal setup provided a good estimate for expression profile at the surface of the ROs but is limited due to their overall diameter (>500 μm). All quantifications of eGFP signals shown in this manuscript were done based on images acquired via standard fluorescence microscopy, which allowed for detection of the overall fluorescence signal.

In the initial experiments, ROs were transduced in 48-well plates and incubation time with viral vector before media change was varied between 1 and 3 days. This, however, did not influence the eGFP signal measured 7 days after transduction (no statistical differences for all tested conditions; Figure S2A). ScAAV2.7m8 showed the strongest overall eGFP expression after 7 days for both day 80 and day 300 ROs, when analyzing all incubation conditions side by side (1–3 days) (Figures 2A and S2A). A substantial fluorescence signal was also observed for scShH10. Here, day 300 ROs showed a slightly stronger signal than day 80 ROs. scAAV2 showed a comparably low expression for both day 80 and 300 ROs; scAAV9 signal was barely detectable in both conditions (Figures 2B and S2). The comparison of signal magnitude at day 1, 3, and 7 post transduction showed distinct kinetics for each AAV serotype. With the exception of scAAV9, a time-dependent increase of expression was observed for scAAV2, scShH10, and scAAV2.7m8. The steepest and overall strongest increase was induced by scAAV2.7m8 (Figures 2C and 2D).

Finally, we assessed if application of AAV vectors affects growth and morphology of the ROs (Figures S2B and S3). As expected, control day 300 ROs did not increase or decrease in size, since most of the cells were already post-mitotic. In contrast, the size of the developmentally young ROs of day 80 still increased around 20% in controls and also in the low-transducing scAAV9. Interestingly, the size of ROs transduced with scAAV2, scShH10, and scAAV2.7m8 at day 80 decreased substantially 7 days after transduction, suggesting either a loss of cells or overall RO integrity (Figure S2B). This was supported by morphological assessment of brightfield images showing that most day 80 ROs transduced with scAAV2 (70%), scShh10 (65%), and scAAV2.7m8 (90%) showed signs of degeneration or RO disorganization (Figures 2A and S3A). In contrast, day 300 ROs did not show any detrimental changes after viral transduction. The observed morphological decline for day 80 ROs was proportional to the duration of viral incubation (Figure S3B). A detailed analysis of day 80 ROs treated for 3 days with the four AAV serotypes, revealed a significant increase of overall cell death (propidium iodide [PI]) and an increased apoptosis (cleaved CASPASE 3) in scAAV2.7m8-treated ROs after 7 days (Figures S3C and S3D). Furthermore, scAAV2-treated ROs showed an increase of PI and cleaved CASPASE-3. scAAV9 and scShH10 did not show substantial increase of both markers. Interestingly, only scAAV2.7m8 showed a strong and significant decrease of KI67 signal, indicating a decrease of proliferation (Figure S3F). This was in line with the morphological data showing a particularly strong tissue disorganization for this serotype.

### The RoC as screening platform for evaluating AAV transduction efficiency

**Figure 3:**
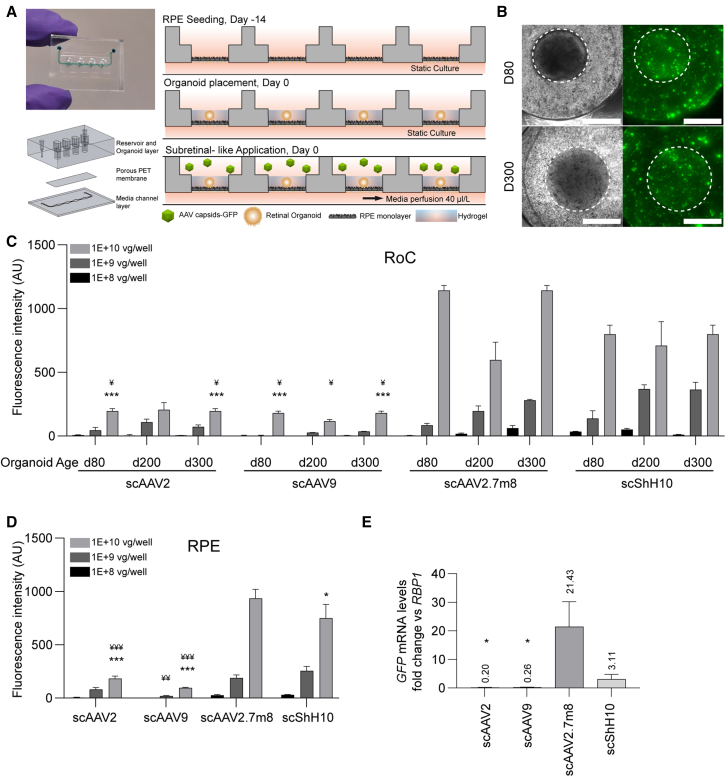
Testing of AAV serotypes in the RoC (A) Adapted RoC design and chip seeding protocol for subretinal AAV treatment. (B) Representative brightfield and GFP fluorescence live imaging. RoC area used for quantification is circled white. Fluorescent images are maximum intensity projections. Scale bars: 500μm. (C) Quantification of the mean eGFP fluorescence after 7 days of culture. Statistical analysis represents the comparison with scAAV2.7m8 (^∗^) and scShH10 (¥) for 1 × 10^10^ virus genomes and the same organoid age. (D) Quantification of the mean eGFP fluorescence in the non-organoid areas of the RoC wells after 7 days of culture. Statistics represent the comparison with scAAV9 (#), scAAV2.7m8 (^∗^), and scShH10 (¥). (E) *eGFP* gene expression for each AAV in the RoC with day 300 ROs and a virus load of 1 × 10^10^ virus genomes per well. Statistics represent the comparison with scAAV2.7m8. (C) n = 3 wells from one RoC, (D) n = 9 wells from three RoCs per condition, and (E) n = 3–4 RO from one RoC per condition, mean + SEM. See also Figures S4 and S5.

The RoC was recently developed to enable a stable co-culture and direct interaction of hiPSC-RPE and -RO while creating a tight outer blood-retina (epithelial) barrier (Achberger et al., 2019). The chip allows for a vascular-like perfusion through a bottom channel and a constant nutrient supply to the tissue-containing well through the epithelium (Figure 3A). This allows different compound application routes: systemic choroid-like application through the bottom channel and subretinal-like application into the wells. The latter allows AAV particles to remain present over a long period of time without nutrient deprivation, mimicking a clinically practiced AAV application.

Each RoC contains four individual wells separated by a permeable membrane from the supply channel below (Figure 3A). To assemble the cellular components of the RoC, RPE cells are seeded in each well on top of the membrane on day −14. After 14 days of static culture, ROs are seeded on top of the RPE and each well is infused with a hyaluronic acid-based hydrogel. Viral transduction occurred upon adding AAVs to the well in a special 27-μL compartment above the hydrogel. Thereafter, the chips were perfused with medium through the bottom channel while keeping the viral-vector-containing upper well static.

To assess the barrier integrity of the RoC after AAV transduction, an RPE chip without RO was transduced with scAAV2 (Figure S4A). We found that less than 1% of initial AAV dose was found in the chip effluent over 24 h of culture, indicating a strong retention of AAV particles in the chip compartment and a tight RPE barrier (Figure S4C). Labeling of F-actin cytoskeleton using phalloidin supported the intactness of the RPE barrier with and without AAV transduction (Figure S4D). This was further substantiated by analyzing the chip well supernatants, showing that around 7% of the viral particles were still present in solution after 6 days of perfusion (Figure S4B). Additionally, we assessed the effect of the hydrogel added to the RoC. We found that it did not influence the viral retention or the tightness of the RPE barrier (Figures S4B–S4D), but decreased the AAV transduction of the RPE (Figure S4E).

Next, ROs of three ages (days 80, 200, 300) were selected and three different doses of viral vector (1 × 10^10^, 1 × 10^9^, 1 × 10^8^ vector genomes per RoC well) were applied to the RoC. After 3 and 7 days, total eGFP fluorescence of RO area and the underlying RPE (referred as RoC area, circled white in Figure 3B) of each AAV serotype was assessed. Seven days post transduction, a clear dose to signal correlation was confirmed for all conditions and AAV serotype (Figure 3C). ScAAV2.7m8 and scShH10 induced the strongest signals throughout the three different RO ages (Figure 3C), significantly outperforming scAAV2 and scAAV9. Both scAAV2 and scAAV9 only induced a weak expression in all RoCs, irrespective of RO age. When assessing the AAV transduction in the non-organoid, solely RPE-cell-containing area, again scAAV2.7m8 and scShH10 led to significantly higher signals than scAAV2 and scAAV9 (Figure 3D). In the following, spatial distribution of the eGFP signal was visualized using confocal imaging (Figure S5A). We found that both RPE and RO contributed to the overall eGFP fluorescence, with the strongest eGFP signal found in the scAAV2.7m8-transduced RoC. This was supported by the assessment of eGFP mRNA levels of RoC-extracted ROs (Figure 3E) in which again scAAV2.7m8 by far induced the highest expression levels with a 21-fold increase (relative to RNA polymerase II) in ROs, followed by ShH10 (3.11), AAV9 (0.26), and AAV2 (0.20). Finally, day 7 to day 3 comparison was used to assess the AAV serotype-specific kinetics (Figures S5B–S5D). These analyzes confirmed the ability to capture distinct transduction profiles of single AAV vectors in the RoC.

### AAV serotypes show wide range of cell tropism in the RoC

**Figure 4:**
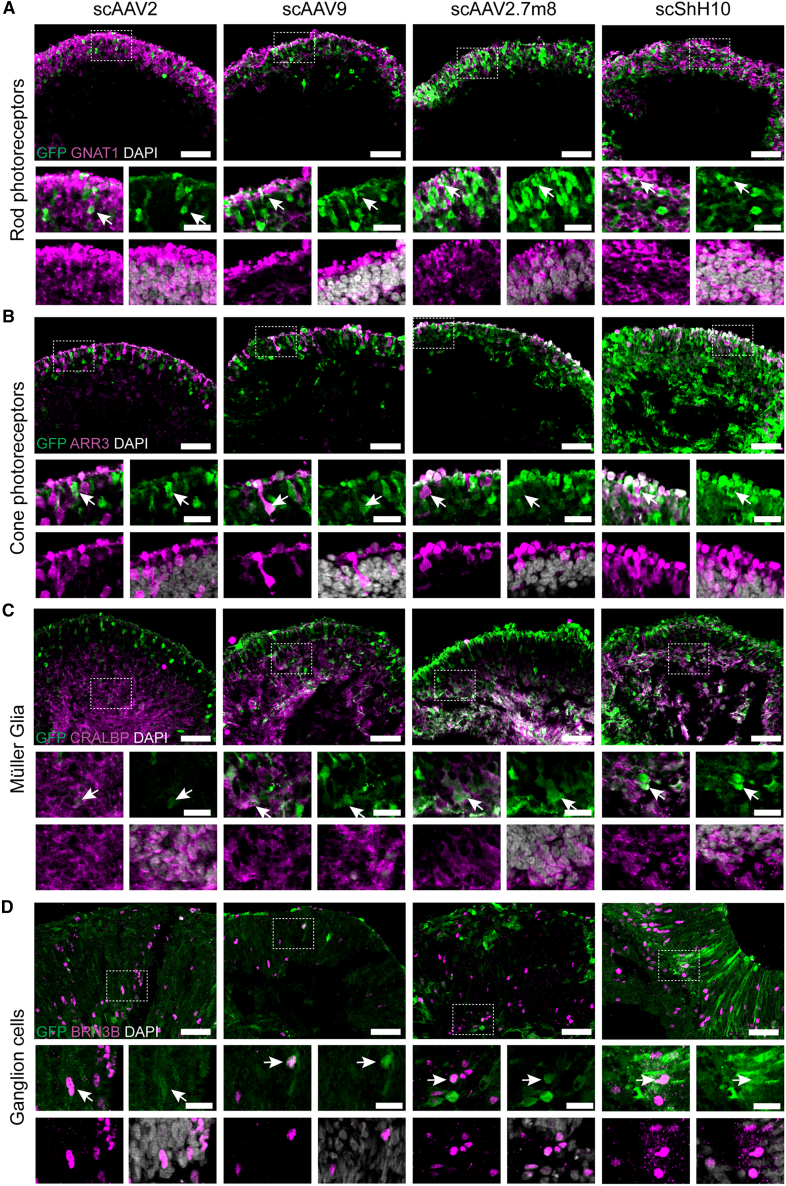
Evaluation of cell tropism in the RoC (A–D) Day 200 ROs in (A)–(C) were transduced with 1 × 10^10^ virus genomes, (D) day 80 ROs were transduced with 1 × 10^9^ virus genomes. (A–D) Vertical cryosections of ROs showing AAV-mediated eGFP expression (green) and cellular co-stainings (magenta): (A) rod transducing (GNAT1, rods), (B) ARRESTIN 3 (ARR3, cones), (C) CRALBP (MüG), and (D) BRN3B (ganglion cells). DAPI: white. Co-stained cells are highlighted with white arrow. Scale bars: 50 μm (large images), 20 μm (small images). See also Figures S6 and S7.

Next, we interrogated cell tropism of different AAV serotypes within the human ROs (Figures 4 and S7). RoCs containing day 200 ROs were transduced with 1 × 10^10^ virus genomes, and day 80 ROs were transduced with 1 × 10^9^ virus genomes. Seven days after AAV application, ROs were harvested, cryosectioned, and co-stained with cell-type-specific markers such as GNAT1 (rod photoreceptors), ARR3 (cone photoreceptors), CRALBP (MüG), and BRN3B (ganglion cells) (Figure 4). Our investigations indicate that all four AAVs were associated with a tropism for rod and cone photoreceptors as well as MüG cells in day 200 ROs (Figures 4A–4C). This cell tropism was recapitulated in day 300 ROs (data not shown). A signal co-localization analysis confirmed that all serotypes had a strong tropism toward cones, rods, and MüG cells (Figure S7A–S7C). As expected, we found that the degree of co-localization between GFP and cell markers was increasing with viral dose. A tropism for ganglion cells, amacrine, and horizontal cells could be found for all four serotypes. Quantitative analysis showed that ganglion cells were efficiently (up to 60%) transduced by all four serotypes (Figure 4D), whereas amacrine (up to 40%) and especially horizontal cells (up to 25%) were only poorly transduced (Figures S7D–S7F). AAV-transduced bipolar cells were found as well but could not be quantified due to a poor signal quality (Figure S6C).

### Screening of next-generation AAVs in the RoC

**Figure 5:**
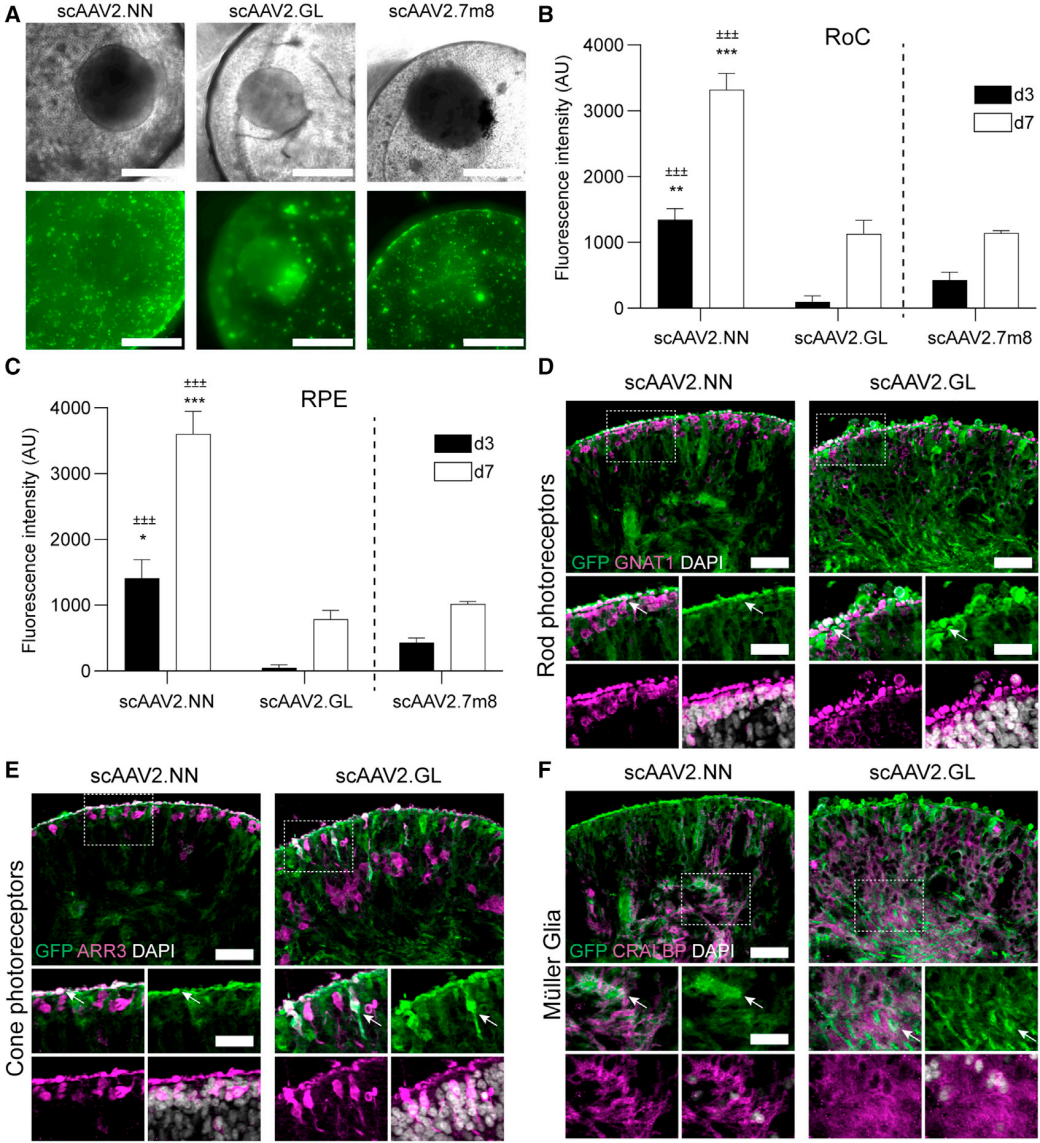
Evaluation of next-generation AAVs in the RoC (A) Representative brightfield and eGFP fluorescence (maximum intensity projection) images of RoCs (1 × 10^10^ vector genomes per well). (B) Quantification of the mean eGFP fluorescence. AAV2.7m8 data (Figures 3 and 4) are depicted as comparison. Statistics represent the comparison with scAAV2.GL (±) and scAAV2.7m8 (^∗^). (C) Quantification of the mean eGFP fluorescence in the non-organoid area of the RoC well. Statistics represent the comparison with scAAV2.GL (±) and scAAV2.7m8 (^∗^). (D–F) Vertical cryosections of day 300 ROs transduced with 1 × 10^10^ virus genomes showing eGFP expression (green) and cellular co-stainings (magenta): (D) rod transducing (GNAT1, rods), (E) ARRESTIN 3 (ARR3, cones), and (F) CRALBP (MüG). DAPI: white. Co-stained cells are highlighted with white arrow. Scale bars: 50 μm (large images), 30 μm (small images). (B and C) n = 3 wells from one RoC for all conditions, mean + SEM. See also Figures S6 and S7.

Aiming to optimize tropism and transduction efficiency, new AAVs are constantly generated. To show the applicability of the RoC as a screening system for novel AAV variants, we applied two recently developed second-generation AAV capsids: AAV2.NN and AAV2.GL (Pavlou et al., 2021). Both variants show highly efficient transduction profiles for various retinal cell types (Pavlou et al., 2021). Here, we analyzed scAAV2.NN and scAAV2.GL together with scAAV2.7m8, which showed most efficient eGFP expression in this study. All AAV capsid variants induced eGFP fluorescence in RO and RPE after 7 days of culture (Figure 5A). Comparison of mean intensities of eGFP signals revealed that scAAV2.NN induced the highest expression, significantly outperforming scAAV2.7m8 (Figure 5B). scAAV2.GL showed a comparable expression strength with scAAV2.7m8 both at day 3 and day 7. The comparison of the non-organoid, RPE area of the respective chips rendered similar results: again, the eGFP signal of scAAV2.NN-transfected RoC wells was significantly higher than the other conditions at both day 3 and 7.

Assessing the cellular transduction profile of the second-generation AAVs in the RoC with day 300 ROs, we found that both AAVs were extremely efficient in transducing rod and cone photoreceptors and MüG cells (Figures 5D–5F and S7B). In contrast, both tested serotypes induced only a poor GFP expression in horizontal cells (Figure S7D). Amacrine cells and ganglion cells were not detected in the assessed day 300 RO.

### The RoC enables assessment of long-term effects of AAV transduction

**Figure 6:**
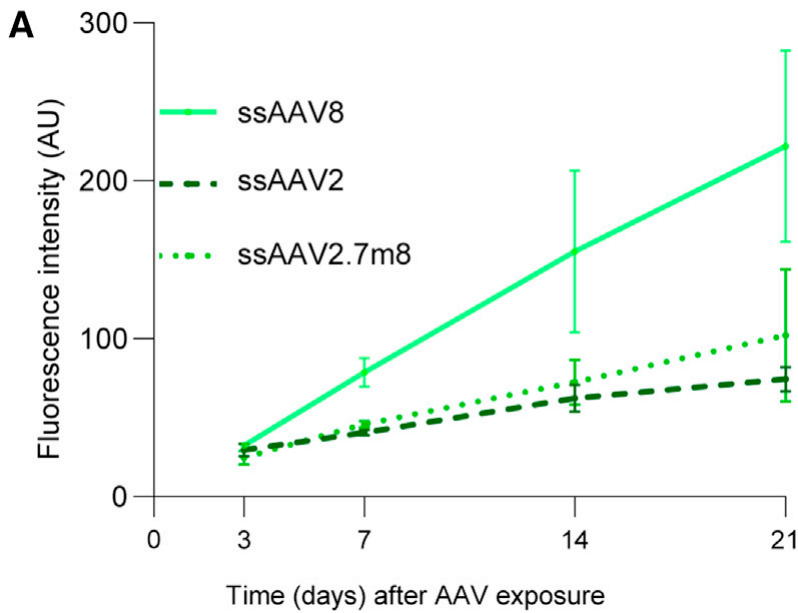
Long-term analysis of AAV-induced expression in the RoC using ssAAV8, ssAAV2, and ssAAV2.7m8 Quantification of the mean eGFP fluorescence in the RoC with day 300 ROs exposed to 1 × 10^10^ virus genomes per well. n = 3 wells from one RoC for all conditions, mean ± SEM.

One major advantage of the RoC is its long-term stability, which allows analysis of the AAV-induced expression over a prolonged period of time. This is of particular importance to mimic development after application, kinetics of viral transcription, but also to gain pharmacokinetic information about vector-based delivery of therapeutics. In the RoC, AAV vectors can be added to the upper well, which can be sealed after application, thus creating an enclosed, stable system nurtured by the medium provided by the vasculature-mimicking bottom channel (cf. Figure 3A). To demonstrate the ability of long-time monitoring, we cultured the RoC for 21 days in the presence of three different viral vectors (ssAAV8, ssAAV2, and ssAAV2.7m8; Figure 6) and monitored eGFP signals after 3, 7, 14, and 21 days. SsAAV2 and ssAAV2.7m8 showed only a faint fluorescence signal at day 7, which slightly increased until day 21 (Figure 6). This is in line with ss vectors being less potent and having a lower-onset kinetic than sc vectors. At the same time, ssAAV8 eGFP showed a strong eGFP signal on day 7 continually increasing over the course of 21 days, resulting in the highest expression of the three tested AAVs. In brief, we could demonstrate that the RoC can be used to continuously monitor transgene production in human retinal cells for an extended period of time.

## Discussion

The advent of human stem cell-derived microphysiological systems paralleled by AAV-based gene therapies maturing into actual therapies led to the obvious and appealing concept of combining these two fast-evolving fields to foster the development of novel AAV vectors and efficient gene therapies. Being immune privileged, highly compartmentalized, with good accessibility and low number of cells to be targeted, the eye is the ideal organ for a gene therapeutic approach. Hence, retinal diseases are a main focus of AAV-based research for which the versatility, availability, and relevance of human ROs hold great value. Previous reports indicated the feasibility and validity of this approach, comparing AAV transduction efficacy on stem cell-derived RPE and photoreceptors (Gonzalez-Cordero et al., 2018) and analyzing mechanisms of cell attachment and their interplay with primary cell-surface receptors (Garita-Hernandez et al., 2020). AAVs were even successfully used to correct congenital retinopathy phenotypes in patient stem cell-derived ROs (Kruczek et al., 2021). Here, we further extended this concept by an in-depth analysis of various capsids linked to retinal gene therapy. The naturally occurring serotypes AAV2, AAV8, and the developed AAV2.7m8 were chosen, as they are currently applied in clinical trials or approved retinal therapy (Buck and Wijnholds, 2020; Shahryari et al., 2019). Furthermore, AAV2.7m8 is a second-generation AAV developed via *in vivo* directed evolution, created and selected for more efficient delivery in ocular gene therapies (Dalkara et al., 2013). AAV9, on the other hand, has shown the ability to efficiently transduce human cells in patients (Shahryari et al., 2019) with a broad cellular tropism when delivered systemically but is not a candidate for retinal gene therapy. In addition to these well-studied vectors, newly developed variants with reported specific tropism or increased efficacy in preclinical models were evaluated (Klimczak et al., 2009; Pavlou et al., 2021). To recapitulate the complex tissue structure of the human retina and physiological drug delivery routes better than in ROs alone, we employed a RoC model. The RoC provides several advantages, including physiological interaction of RO and RPE, compartmentalization, and vasculature-like perfusion. The latter aspect not only enables a constant nutrient supply mimicking choroidal vasculature but also provides the ability to deliver compounds in a subretinal-like manner. Thus, AAV particles can be added to the upper well and are retained by a tight RPE barrier. In addition, the optical accessibility and stability of the RoC provides the opportunity for extended *in situ* live-cell imaging.

First, we confirmed the efficacy of our AAV vectors in an animal model *in vivo*. Therefore, we applied the well-described serotypes ssAAV2, harboring a ss eGFP, scAAV2, scAAV2.7m8, and scShH10, harboring an sc sequence eGFP, all under control of CMV promotor intravitreally to adult mice. Obtained data confirmed the expected increased expression of scAAV2s compared with ssAAV2 (McCarty et al., 2001) and the superior transduction efficacy of scAAV2.7m8 compared with scAAV2 (Khabou et al., 2016). The published preferential tropism for MüG in rodents of the ShH10 variant, a vector generated from an AAV6 parent serotype shuffled (ShH) library (Klimczak et al., 2009), could also be reproduced and was especially prominent at lower vector doses.

Second, we transferred these vectors together with the AAV9 vector to iPSC-ROs of different ages in order to assess the impact of maturation on AAV transduction efficacy. AAV9 was added as it exhibited potent transduction after subretinal injections to rodents (Allocca et al., 2007) but was unsuccessful to transduce retinal cells after intravitreal injections *in vivo* (Lee et al., 2019). The application of AAV9 to human retinal explant cultures from the subretinal side showed a rather low transduction efficacy (Wiley et al., 2018). In our study, scAAV9 eGFP signals were barely above the detection limit both in day 80 and day 300 ROs up to 7 days post infection. In comparison, ROs transduced with scAAV2, scAAV2.7m8, and scShH10 displayed augmented eGFP signals of which scAAV2.7m8 promoted the strongest response. Albeit using different protocols for iPSC-RO generation and AAV application, previous studies also reported poor efficacy of scAAV9 (Gonzalez-Cordero et al., 2018) and higher potency of AAV2.7m8 versus AAV2 vector (Garita-Hernandez et al., 2020). The authors of these studies indicated the lack of efficacy due to the developmental stage of their ROs (13 weeks) with missing outer segments. However, we tested AAV9 on ROs with the age of up to 42 weeks with formed inner and outer segments, suggesting that other mechanisms might be responsible for the lack of transduction.

After validating the effects of the AAV vectors on ROs, the same vector panel was applied to the RoC. Here, scAAV2 transduced retinal cells as well as the RPE. Interestingly, the RPE transduction efficiency was reduced in the presence of a hyaluronic acid-based hydrogel. We interpret this as a physiological feature of the RoC, since the hydrogel was intended to mimic the hyaluronic acid-rich subretinal space. The ability of scAAV2 to transduce RPE and retinal cells is in line with the capsid properties shown in rodent models (Auricchio et al., 2002) and essential for the therapeutic benefit of Luxturna in patients. Aside from AAV2, a more significant eGFP expression in RPE and retinal cells was induced by the scAAV2.7m8 and scShH10 vector variants. Remarkably, the reported cellular tropism of ShH10 in rodents toward MüG cells did not translate to the RoC. However, in the human RoC setting, the ShH10 vector shows a high potency to transduce rods, cones, and RPE with comparable potency with the AAV2.7m8. This is in line with results from Gonzalez-Cordero et al. (2018) testing ShH10 using human iPSC-ROs. We also detected the extension of ShH10 transduction to ganglion and horizontal cells, which had not been analyzed in previous studies. Notably, this non-selective tropism was observed in all virus doses tested (e.g., 1 × 10^8^ vector genomes per RoC well; Figure S7). Overall, the acquired immunofluorescence data suggest that there is no relationship between dose and cell tropism and that the tropism found in rodents does not fully translate to models based on iPSC-derived human cells. This raises concerns about whether the reported MüG cell tropism can be translated to the human retina. Additional data from this vector in higher species are unfortunately lacking but might give a more complete picture of this vector's capacities.

The highest measured signals were obtained from RoC treated with scAAV2.7m8. The AAV2.7m8 capsid variant was selected from an AAV2-based peptide insertion library and has a higher potency to transduce murine and NHP retinae (Dalkara et al., 2013). Therefore, this variant was utilized as the clinical phase II candidate vector for intravitreal injection aiming for the treatment of patients with wet age-related macular degeneration and diabetic macular edema (Grishanin et al., 2019) currently in clinical phase II. In unison with the convincing data compiled for the AAV2.7m8 vector variant, our RoC model delivers supportive data for the capabilities of this newly generated AAV vector, particularly to efficiently transduce the main retinal cell types, including photoreceptors, MüG, and ganglion cells.

In addition, we interrogated whether and how newly developed AAV vectors selected for retinal application would perform in the RoC. Therefore, we applied the two recently published variants AAV2.NN and AAV2.GL (Pavlou et al., 2021). These AAV2-based heptamer insertion mutants were reported as potent transducers of retina in mice, dogs, NHPs, and human explants (Pavlou et al., 2021). To assess their performance in the RoC, AAV2.NN and AAV2.GL were compared with scAAV2.7m8, which served as an intra-experimental control. While scAAV2.GL showed comparable expression with scAAV2.7m8, scAAV2.NN significantly surpassed both, showcasing how the RoC potential can be leveraged in order to screen and detect increased potency of novel AAV variants for transduction of human retinal tissue. Furthermore, both scAAV2.GL and scAAV2.NN showed a superior efficiency to transduce cones, rods, and MüG cells (Figure S7).

Taken together, our study reveals for the first time the potential of a complex microphysiological system as a translational model for testing of AAV vectors in a clinical-like setup. We could show that the RoC offers the ability to directly compare transduction efficacy and cellular tropisms using a subretinal-like application route, which is one of the two mainly used application routes in the clinical context. Here, an important future implementation of the RoC will be to mimic the intravitreal application currently limited by the spheroidal nature of the RO and the absence of an accessible inner limiting membrane. Furthermore, several open questions need to be addressed in the future. A major point is the existence of inter-donor variability concerning efficiency and tropism (Ling et al., 2016). For AAVs, the tropism depends on the glycan and proteinaceous AAV receptor frequency and composition (Pillay and Carette, 2017). Although very little is still known on the transduction properties of AAV capsids of the retina after subretinal injections in human patients, transduction profiles are very consistent between single animals of one species and also between different species. AAV2 tropism to RPE, for example, was first identified in mice, is translatable to a RPE65 dog model, and now shows clinical efficacy with Luxturna. To our knowledge, Wiley et al. (2018) is the only study reporting issues in intra-donor variability in retinas from human donors (only four donors) after subretinal-like injections. Therefore, a broad, comprehensive assessment of donor-related effects on transduction efficacy after subretinal injections in different species, and especially in human, needs to be performed, an effort for which the presented RoC provides an invaluable tool as presented in this study.

In the context of the RoC, the presence of a choroidal vascularization and integration of immune components need to be further explored. The implementation of these aspects is possible using OoC technology (Cipriano et al., 2021) but was not included in this study to reduce the system complexity. Although the direct effect of the vascularization component on AAV transduction and tropism might be small, a fully vascularized arrangement could possibly result in higher retention time of the AAV in the retinal compartment and the interaction with immune cells could strongly affect AAV efficacy and safety. Overall, we anticipate that the RoC and its future developments will be an important asset in the development and safety assessment of new vector candidates for future retinal gene therapies.

## Experimental procedures

### AAV vectors

We used AAV2, AAV8, AAV9, AAV2.7m8 (Dalkara et al., 2013), ShH10 (Klimczak et al., 2009), and AAV2.NN, AAV2.GL (Pavlou et al., 2021) with the ss expression cassettes ss-CMV-eGFP-SV40poly(A), ss-CMV-anti-FITC, or sc-CMV-eGFP-SV40poly(A) (Strobel et al., 2020), flanked by AAV2-derived inverted terminal repeats. AAV vectors were prepared as in Strobel et al. (2019): HEK 293-H cells were cultured in DMEM + GlutaMAX-I + 10% FCS (Thermo Fisher Scientific) and transfected as previously described (Strobel et al., 2015). AAV purification via polyethylene glycol precipitation, iodixanol gradient, ultrafiltration, and sterile filtration was conducted as previously described (Strobel et al., 2019).

### Virus titer measurement via digital droplet PCR

AAV genomic titers were determined by digital droplet PCR (primers Table S2) using QX200 system (Bio-Rad, United States). Viral DNA was prepared with ViralXpress (Merck Millipore, United States). Droplets were generated using the Droplet Generator (Bio-Rad, United States). X50s PCR Mastercycler (Eppendorf, Germany) was used with initial denaturation step 10 min at 95°C, followed by 40 cycles of 30 s at 95°C with annealing for 1 min at 60°C. AAV genomic titers were analyzed with Droplet Reader and the QuantaSoft software (Bio-Rad, United States).

### Animal experiments

Experiments conducted with 9–12-week-old C57BL/6J female mice (Charles River, Germany) according to the German Welfare Law and the GV-SOLAS guidelines (Dülsner et al., 2017) were approved by local animal welfare authority (Regierungspräsidium Tübingen, Germany). Mice were anesthetized with 3.5% isoflurane and 1 μL of AAV suspension was injected transsclerally into the vitreous using a 34-G canula (WPI, United States). Animals were sacrificed under anesthesia by cervical dislocation 3 weeks after injection and eyes were harvested in 4% paraformaldehyde (PFA) for immunohistochemistry or liquid nitrogen for RT-qPCR.

### RoC fabrication

Fabrication of the RoC was conducted as described previously (Achberger et al., 2019), with a slight modification of the top layer design to increase the volume of the tissue compartments to 27 μL.

### Cell culture

The iPSC line INDB-5-1 used in this study was derived from hair keratinocytes of a healthy male donor as previously described (Linta et al., 2012): The line was generated using a lentiviral polycistronic vector (Warlich et al., 2011) (Figure S1; antibodies in Table S1; qPCR primers in Table S2). Absence of karyotypic abnormalities was confirmed using the hPSC Genetic Analysis Kit (STEMCELL Technologies, CA). iPSCs were cultured on Matrigel (hESC-qualified, BD Biosciences, United States)-coated plates with FTDA medium (Frank et al., 2012). Cells were passaged every 6–7 days using Dispase (STEMCELL Technologies, CA). All procedures were in accordance with the Helsinki Convention and approved by the Ethical Committee of the Eberhard Karls University Tübingen (no. 678/2017BO2). The control person gave his written consent.

Differentiation and culture of hiPSC-ROs, derivation of RPE cells as a product from RO differentiation, and assembly and culture of the RoCs was conducted as previously described (Achberger et al., 2019). In contrast to the previous reported protocol, RPE was cultured for 2 weeks prior to the loading of ROs with daily medium change. Quality of RO and RPE was ensured by morphological assessment, which included for ROs monitoring of typical layering, timepoint-dependent presence of segments as well as absence of RPE patches, and for RPE confirmation of hexagonal shape as well as pigmentation in both dish and RoC. Solely RoCs perfused sufficiently throughout the culture period were included.

### AAV treatment

#### Dish culture

On day −1, one RO was placed in each well of non-adherent 48-well plates in 300 μL of BRDM (DMEM/F12 [3:1]) with 2% B27 (w/o vitamin A), 1× non-essential amino acids, 1× antibiotics-antimycotics + 10% FBS (all Thermo Fisher Scientific, United States). On day 0, AAVs were thawed and added via a 33.3% medium change. After 1, 2, or 3 days of AAV exposure, a medium change was performed, including washing with Dulbecco's phosphate-buffered saline (PBS; no calcium, no magnesium, Thermo Fisher Scientific, United States). An additional medium change was done on day 5. For labeling with PI (Thermo Fisher Scientific, United States), ROs were incubated with 2 μg/mL in BRDM + FBS medium for 15 min.

#### RoC

The AAV treatment consisted of replacing the volume of medium with the AAV solution containing the desired virus genome number. The volume added was between 1 and 8 μL. The final volume of the tissue compartment was kept to 27 μL, defined by the chip design.

### Immunohistochemistry

Whole eyes were fixed in 4% PFA and paraffin embedded. Sections 3 μm thick were deparaffinized and rehydrated by serial passage through changes of xylene and graded ethanol for immunohistochemistry (IHC) staining. Antigen retrieval was performed by incubating the sections in Leica Bond Enzyme solution (Leica Biosystems, Germany) for 5 min. Sections were incubated (30 min, room temperature [RT]) with an anti-GFP antibody (Table S1) in Primary Antibody Diluent (Leica Biosystems, Germany). Bond Polymer Refine Detection (Leica Biosystems, Germany) was used for detection (3,3′diaminobenzidine [DAB] as chromogen) and counterstaining (hematoxylin). Staining was performed on the automated IHC Bond-III platform (Leica Biosystems, Germany).

RoCs were disconnected from the perfusion, washed with PBS, and fixed with 4% Histofix (Carl Roth, Germany) using mild agitation (1 h, RT). After fixation, the RoCs were washed with PBS and stored at 4°C. One day before embedding, ROs were retrieved from RoCs by flushing wells with PBS. The collected ROs were kept in 30% sucrose (in PBS) overnight, embedded in cryomolds using Tissue-Tek OCT (Sakura Finetek, United States), and stored at −80°C until cryosectioning with a cryostat (14-μm slices, CM 3050 S Cryocut, Leica Biosystems, Germany), mounted on Superfrost microscope slides (Thermo Fisher Scientific, United States). Before staining, slides were rehydrated in PBS for 15 min and incubated in blocking solution (10% donkey serum in PBS + 0.2% Triton X-) for 1 h, with primary antibodies (Table S1; diluted in blocking solution) overnight at 4°C and with secondary antibodies (Abcam, UK; in 1:1 blocking solution:PBS) for 2 h at RT. Mounting was performed with ProLong Gold Antifade Reagent with or without DAPI (Thermo Fisher Scientific, United States). Residual antibodies were removed by washing thrice with PBS for 3 min after each antibody incubation. Where indicated Hoechst 33342 (1:2,000, Thermo Fisher Scientific, United States) was added to the last washing step.

### Gene expression analysis

For RNA isolation, samples were homogenized in 900 μL of RLT buffer (Qiagen, Germany), using a Precellys 24 homogenizer and ceramic bead tubes (VWR, United States) at 6,000 rpm for 30 s. Three-hundred and fifty microliters of phenol-chloroform-isoamyl alcohol (Sigma-Aldrich, United States) was added to 700 μL of homogenate in a phase lock gel tube and mixed. Following centrifugation for 5 min at 16,000 × *g*, 350 μL of chloroform-isoamyl alcohol (Sigma-Aldrich, United States) was added and mixed again. After 3 min of incubation at RT and centrifugation for 5 min at 12,000 × *g*, the upper phase was collected and pipetted into a deep-well plate placed on dry ice. After processing of all samples, DNA and RNA were purified, using the AllPrep DNA/RNA 96 kit (Qiagen, Germany) as per instructions. Integrity of RNA was analyzed by BioAnalyzer (RNA integrity number >6). AAV vector genomes were measured using extracted DNA and a standard curve generated by serial dilutions of the respective expression plasmid. Taqman runs were performed on an Applied Biosystems ViiA 7 Real-Time PCR System (Thermo Fisher Scientific, United States). For gene expression analysis, equal amounts of RNA were reversely transcribed to cDNA using high-capacity cDNA RT kit (Applied Biosystems, Thermo Fisher Scientific) as per instructions. qRT-PCR reactions were then set up using RT-PCR kit (Applied Biosystems, Thermo Fisher Scientific). Primers used are listed in Table S2.

The RoC was disconnected from the perfusion and washed with PBS through the medium channel and on each well. The bottom layer and the membrane were removed for collection of the RPE using a scalpel. The RO within the hydrogel was collected in 500 μL of PBS. Dry samples were shock frozen in liquid nitrogen. RNA was isolated by pelleting cells, followed by lysis in 350 μL of RLT buffer and purification using the RNeasy mini kit (Qiagen, Germany) according to manufacturer’s instruction. Primers used are listed in Table S2.

### Microscopy

Microscopic assessment of mice sections was conducted with a Zeiss AxioImager M2 microscope and ZEN slidescan software (Carl Zeiss, Germany). Plate-cultured ROs and tile images of RPE chip wells were imaged with a Zeiss Axio Imager.Z1 (Carl Zeiss, Germany). RoCs were imaged with a Leica DMi8 (LEICA Microsystems, Germany). Cryosectioned ROs were imaged using an Imager.M2 Apotome1 (Carl Zeiss, Germany). RoC confocal images were shot using a Zeiss LSM 710.

### Image analysis

Fluorescence quantification of plate-cultured ROs (Figure 2) was conducted using a macro (available at https://github.com/kachberger/Achberger-Stem-Cell-Reports-2021) in ImageJ (imagej.nih.gov). Briefly, the macro determined the RO-covered area using a threshold on the brightfield image and the plugin Analyze Particles. The mean gray values of the selected area were then analyzed in the respective eGFP or PI channel using the Measure plugin of ImageJ. To analyze cryosections (Figures S3D–S3F), the entire organoid area, and for RPE chips (Figure S4E), the well area was analyzed using the Measure plugin. For background values of individual channels, areas without specific signal were selected.

For fluorescence quantification in the RoC, ImageJ was used to generate the projection (sum) for both brightfield and GFP channels. The area of each RO was selected manually in the brightfield image. The RPE area was then selected by selecting the whole well area excluding the area that is covered by the RO. The mean gray values were quantified within this area using the Measure function of ImageJ. All fluorescence values were adjusted for background. For both ROs and RoC, the selection areas allowed us to monitor the average diameters and evaluate possible morphological changes.

Quantification of marker co-localization (Figures S7A–S7C) was performed on apotome stack images using a custom ImageJ macro (available at https://github.com/kachberger/Achberger-Stem-Cell-Reports-2021). Briefly, a defined threshold for each channel and stack (for all images with the same marker the same value was selected) is selected and a binary image created. Binary images of GFP and retinal cell marker channels were added up (“AND” setting; ImageJ Image Calculator tool). The sum of intensity values of all stacks of the merged image (cell marker+GFP+) was then divided by the sum of intensity values of all stacks of the cell marker image (cell marker+), representing the proportion of cell marker signal positive for GFP.

Quantification by cell counting (Figures S7D–S7F) was performed by manually counting double-positive (cell marker+GFP+) and cell marker+ cells.

### Statistics

RO experiments and RoC runs were performed once. Each tissue-containing well was considered one independent experiment. Statistical analysis was performed with GraphPad Prism 9.0 (Graphpad Software, United States) and statistical testing was performed using Student's t test (Figure S4E), one-way ANOVA with Bonferroni post hoc test (Figures 1D, 3E, and S3), and two-way ANOVA with Bonferroni post hoc test (Figures 1A, 2B, 3C, 3D, 5B, 5C, and S2A). ^∗^p < 0.05, ^∗∗^p < 0.01, ^∗∗∗^p < 0.001.

## Author contributions

Conceptualization, P.L., M.D., C.S., S. Kauschke, S. Kreuz, M.C., T.L., S.M., U.M., A.K., S.L., and K.A.; methodology, M.D., C.S., M.C., K.A., A.K., and J.C; investigation, M.C., M.D., J.R., L.M., V.C., S.P., N.P., B.S., S.M.H., and S.C; writing, K.A., M.D., P.L., S.L., M.C., A.K., and C.S.; resources, T.L., U.M., P.L., and S.L.; supervision, P.L., S.L., S. Kreuz, M.D., and U.M.

## Conflict of interests

M.D., C.S., B.S., T.L., S. Kauschke, S. Kreuz, and U.M. are employees of Boehringer Ingelheim Pharma GmbH & Co. KG. K.A., S.L., and P.L. hold a patent related to the technology presented in the manuscript (WO2019068640A1).

## Supplemental information

Document S1. Supplemental experimental procedure, Figures S1–S7, and Tables S1 and S2

Document S2. Article plus supplemental information
