# Supplementary material for: Human stem cell-based retina on chip as new translational model for validation of AAV retinal gene therapy vectors
Source: Stem Cell Reports. 2021 Sep 14;16(9):2242–56. doi: 10.1016/j.stemcr.2021.08.008 (PMC8452599; doi:10.1016/j.stemcr.2021.08.008)
Supplement: Document S1. Supplemental experimental procedure, Figures S1–S7, and Tables S1 and S2 [file mmc1.pdf]

Stem Cell Reports, Volume *16*

Supplemental Information

Human stem cell-based retina on chip as new translational model for validation of AAV retinal gene therapy vectors

Kevin Achberger, Madalena Cipriano, Matthias J. Düchs, Christian Schön, Stefan Michelfelder, Birgit Stierstorfer, Thorsten Lamla, Stefan G. Kauschke, Johanna Chuchuy, Julia Roosz, Lena Mesch, Virginia Cora, Selin Pars, Natalia Pashkovskaia, Serena Corti, Sophia-Marie Hartmann, Alexander Kleger, Sebastian Kreuz, Udo Maier, Stefan Liebau, and Peter Loskill

Supplemental Information

Supplemental Material and Methods

RPE Integrity experiments

To test the integrity of the RPE layer, RPE containing chips were cultured for one week statically with BRDM medium. Subsequently, instead of adding ROs embedded in HyStem C- hydrogel (ESI Bio, United States) solely the hydrogel was added. Each chip well was then transduced with 1E+10 scAAV2 virus genomes per well and cultured with perfusion as described above. On day 1 and day 6, the effluent of the prior 24 hours was collected. Supernatant was collected at day 6 by pipetting off all liquid from the individual chip wells. GFP images were taken on day 7. AAV vector DNA in the supernatant and effluent were extracted using the DNeasy Kit (Qiagen, Germany) according to the manufactures protocol. TaqMan run using AAV vector primers (sequences see paragraph “Virus titer measurement via digital droplet PCR”) was performed on an Applied Biosystems One Step Plus (ThermoFisher Scientific, United States) system. AAV vector genomes were quantified using a standard curve generated by a serial dilution of the scAAV2 stock in medium used in the experiment.

Supplemental figures

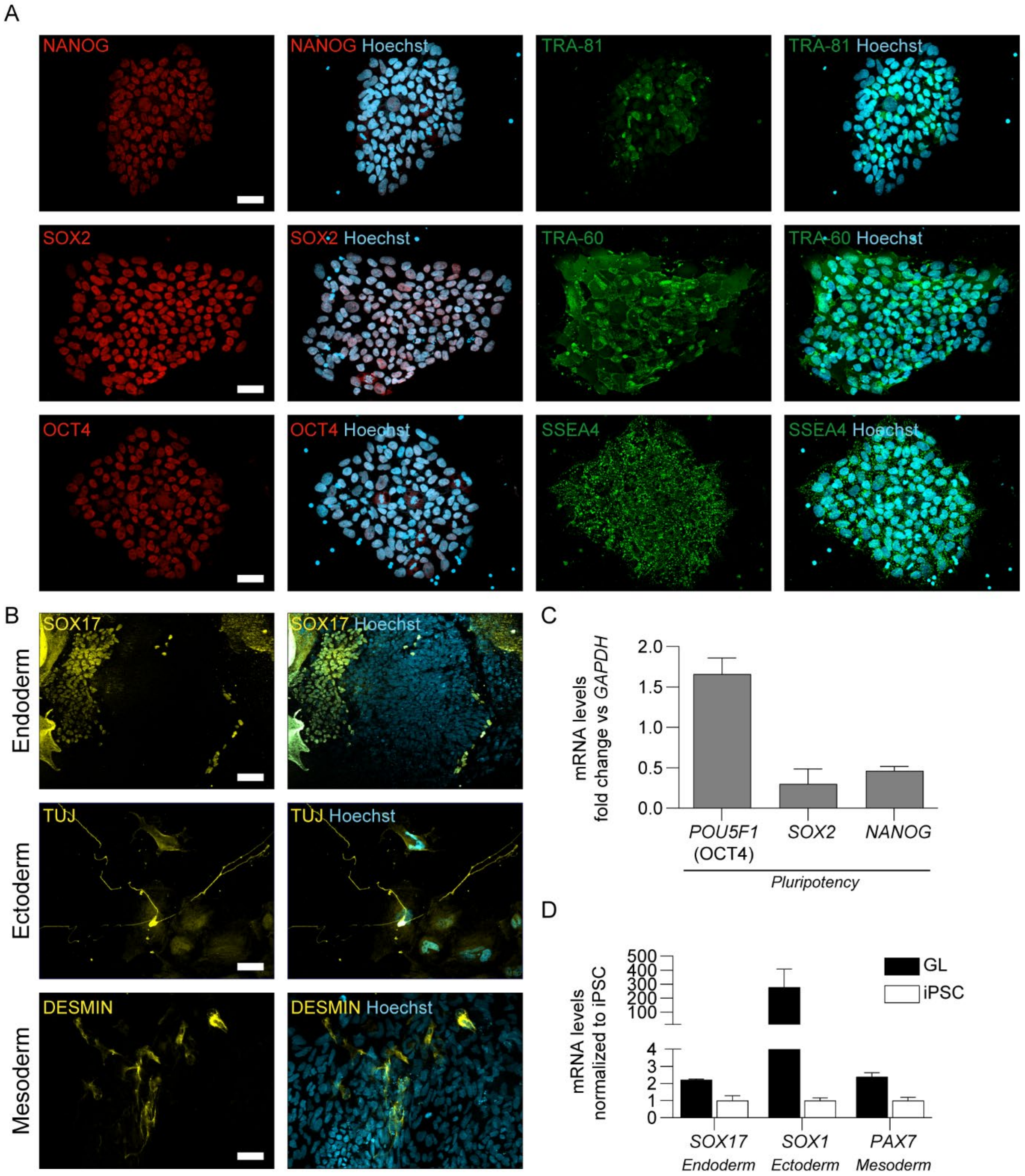

Figure S1. Characterization of the iPSC line INDB-5-1 used in this study.
(A) Immunofluorescence stainings of iPSC showing the pluripotency markers SOX2, OCT4, TRA-60 and SSEA4. Hoechst: light blue. Scale bars: 50µm (B) Immunofluorescence stainings of a germ layer differentiation analyzed at day 15. One marker per germ layer is shown. SOX17: endoderm, β-III-TUBULIN (TUJ): ectoderm and DESMIN: mesoderm. Hoechst: light blue. Scale bars: 50µm (C) Gene expression analysis of pluripotency marker in the iPSC line INDB-5-1. mRNA levels are shown relative to the housekeeping gene *GAPDH*. n=2 iPSC wells per condition, Mean + SEM. (D) Gene expression of a germ layer differentiation with the iPSC line INDB-5-1 anaylzed at day 15. mRNA levels were normalized to the housekeeping gene *GAPDH* and are depicted relative to iPSC mRNA levels. n=2 differentiation wells per condition, Mean + SEM.

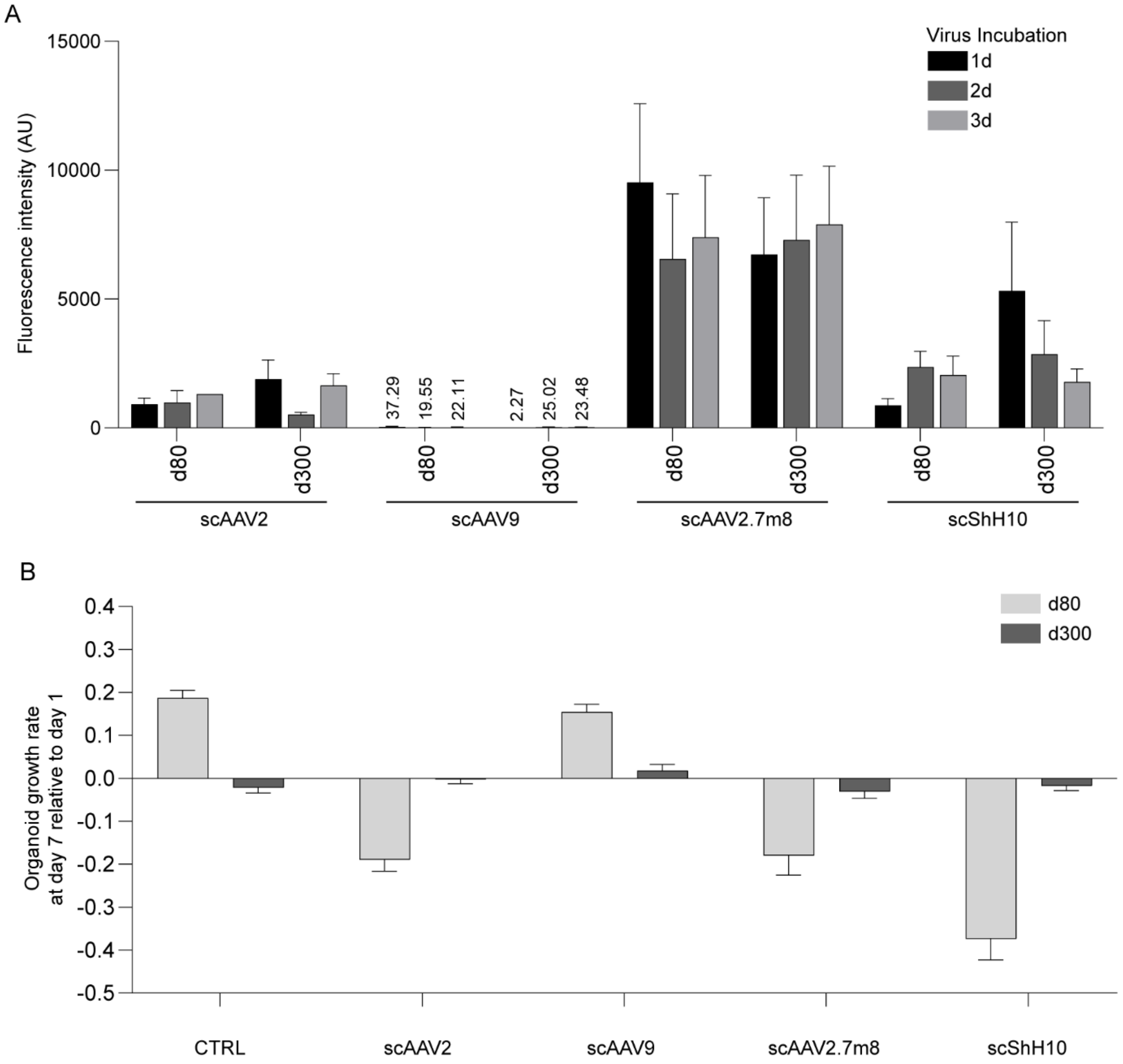

Figure S2. eGFP expression and growth of AAV treated retinal organoids, Related to Figure 2.
(A) Quantification of the mean eGFP fluorescence in day 80 and day 300 retinal organoids exposed for 1, 2 or 3 days to the respective AAV (1E+10 virus genomes/well) and quantified after 7 days. Numbers above the bars give the mean eGFP fluorescence for the respective conditions. There is no statistical difference between the 3 incubation times for all conditions (Two-Way ANOVA with Bonferroni post-hoc test). n=5-6 separate RO per condition, Mean + SEM. (B) Graphs shows relative retinal organoid size comparing day 7 and day 1 after AAV transduction. Controls were untransfected. n=6 separate RO for control and n=18 separate RO for AAV treated organoids, Mean + SEM.

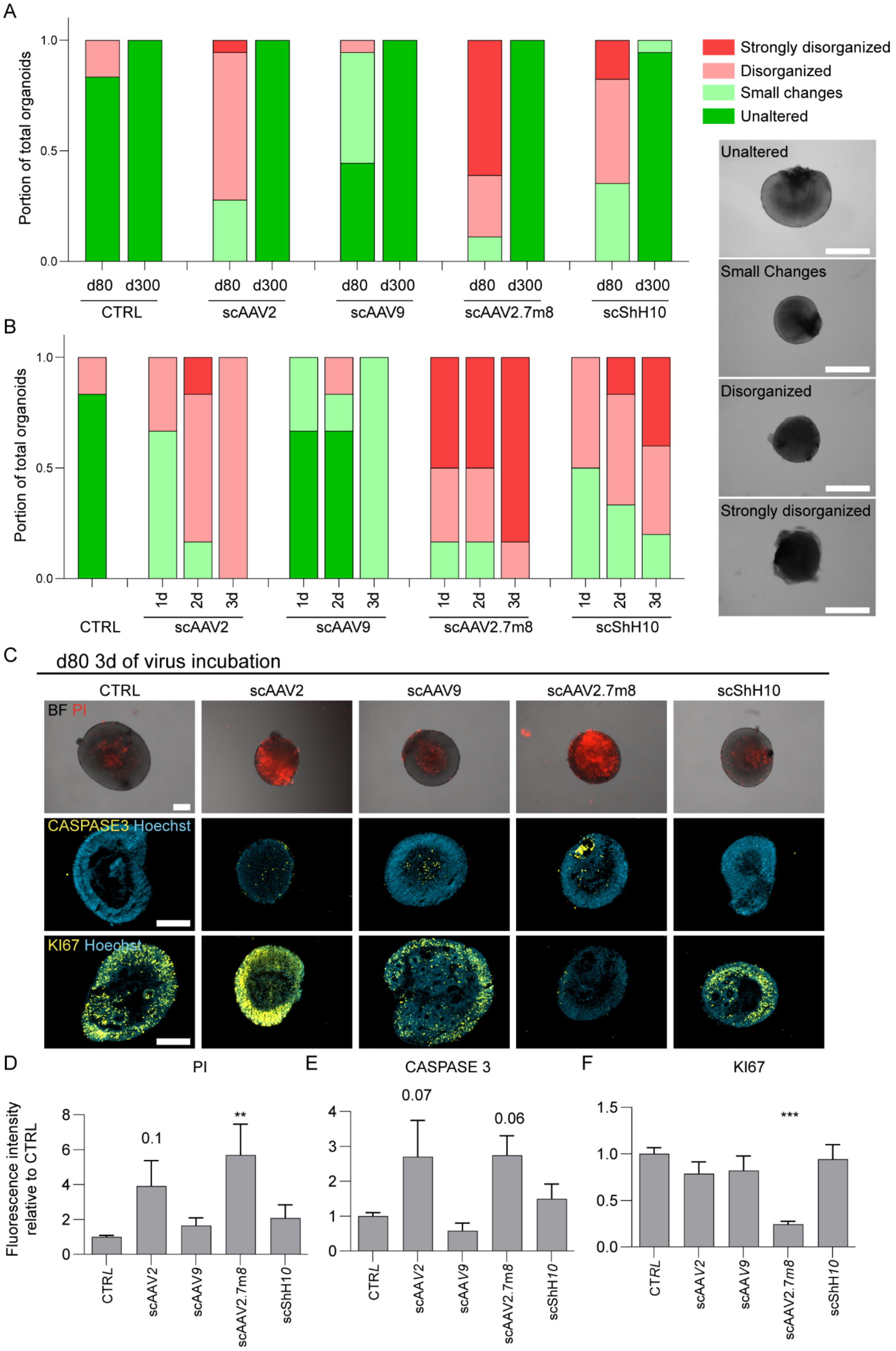

A
Portion of total organoids
1.0
0.5
0.0
d80 d300
CTRL
scAAV2
scAAV9
scAAV2.7m8
scShH10
Strongly disorganized
Disorganized
Small changes
Unaltered
Small Changes
Strongly disorganized
B
1d 2d 3d
C
d80 3d of virus incubation
BF PI
CASPASE3 Hoechst
KI67 Hoechst
D
PI
Fluorescence intensity relative to CTRL
0.1
**
E
CASPASE 3
0.07
0.06
F
KI67
***

Figure S3. Integrity assessment of ROs after 7 days of AAV transduction, Related to Figure 2.
(A) Morphological assessment of day 80 and day 300 retinal organoids for non-treated control (CTRL) and transduced with the AAV serotypes. Criteria were as indicated in the legend showing exemplary images. Graph shows the proportion of the total assessed retinal organoids for each morphological score. n=6 separate RO for controls and 17-18 separate RO for treated conditions. Scale bars: 500μm (B) Organoid morphological of day 80 retinal organoids discriminated by incubation time (1 day, 2 days and 3 days).n=5-6 separate RO for all conditions. (C) Immunofluorescence images of day 80 organoids that have been incubated for 3 days with 1E+10 vector genomes of the respective AAVs or were not transduced (CTRL). Images were taken on day 7 after transduction. First row shows cell death labelling (Propidium iodide, PI) merged with a brightfield image (BF). Second row shows apoptosis staining (Cleaved CASPASE 3); third row proliferative cells (KI67). Hoechst = light blue. Scale bars: 200 μm. (D) Quantification of PI, cleaved CASPASE 3 and KI67 stainings. n=4 separate RO per condition. Mean + SEM.

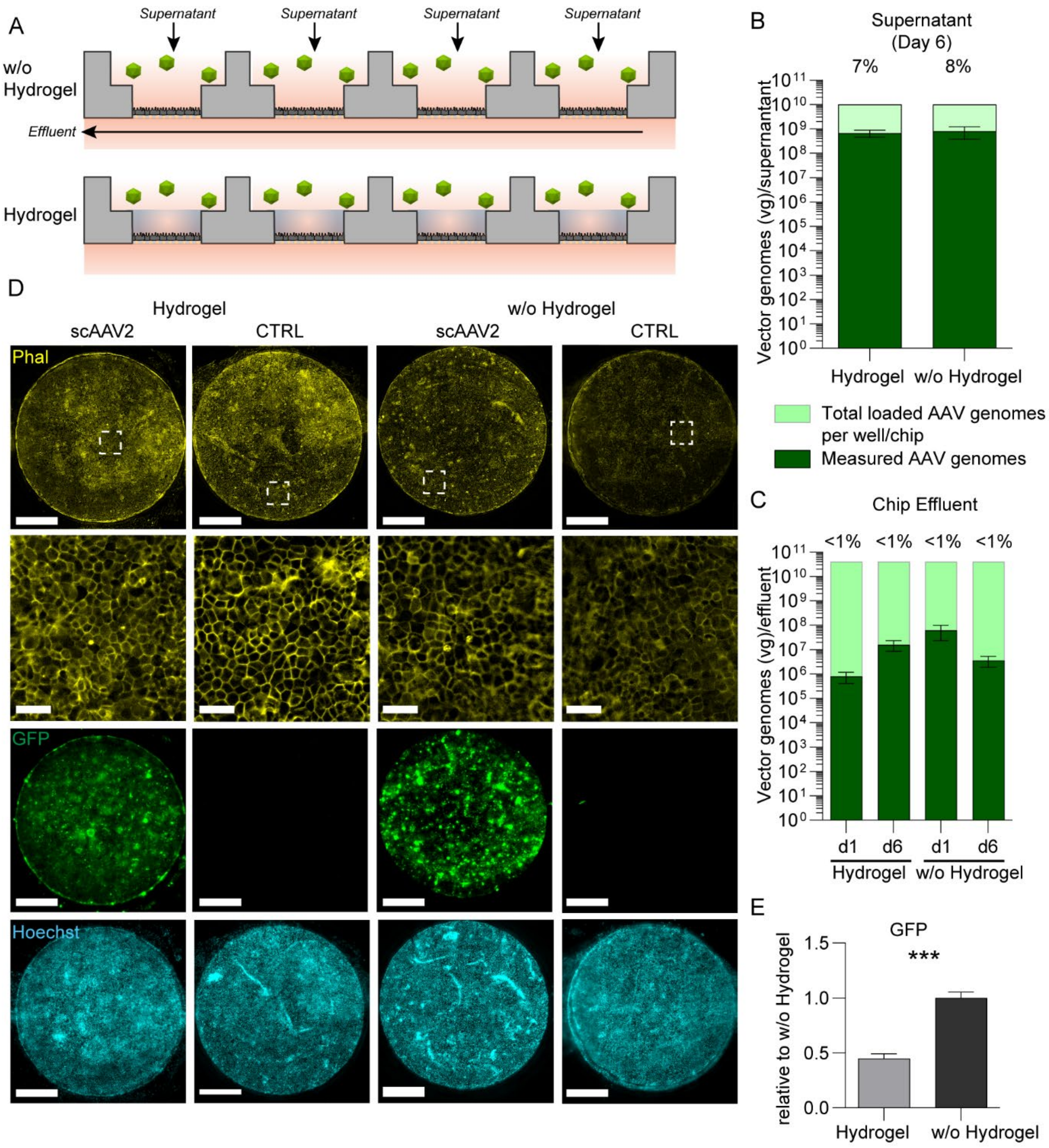

Figure S4. Assessment of RPE barrier integrity in a RoC transduced with scAAV2. ***Related to Figure 3.*** (A) Schematic representation of the experimental setup. RoC containing RPE cells were either cultured covered with or without hydrogel (“Hydrogel” and “w/o Hydrogel” respectively). Both chip types were transduced with 1E+10 vector genomes scAAV2 per well on day 0. On day 1 and day 6, effluents were collected for 24 hours. Supernatants of individual wells were collected at day 6. (B) Quantification of scAAV2 vector genome in the supernatant on day 6 in chips with and without hydrogel. Values are shown as relative amount of vector genomes to the loaded AAV genomes on day 0 (1E+10 vector genomes per well). Y-axis represents a log10 scale. n=5 Chip well supernatants from 3-4 separate chips per condition. Mean ± SEM.(C) Quantification of scAAV2 vector genome in the effluent on day 1 and day 6 of chips with and without hydrogel. Values are shown as relative amount of vector genomes 6 to the loaded AAV genomes on day 0 (4E+10 vector genomes per chip). Y-axis represents a log10 scale. n=3-4 chip effluents from 3-4 separate chips per condition. Mean ± SEM. (D) Representative images of chip wells at day 7 comparing RPE chips with and without hydrogel as well as scAAV2-treated and untreated RPE chips. Cells were labelled using Phalloidin Alexa Fluor 647 (First row, Phal, yellow) showing junctions and cytoskeleton. Second row shows a magnified area as indicated in the first row. Third row shows GFP signal caused by scAAV2 transduction. Fourth row shows Hoechst (light blue). Scale bars: 500 μm and 50 μm (magnified images). (E) Quantification of GFP signal comparing RPE chips with and without hydrogel. n=12-16 chip wells from 3-4 separate chips per condition. Mean + SEM.

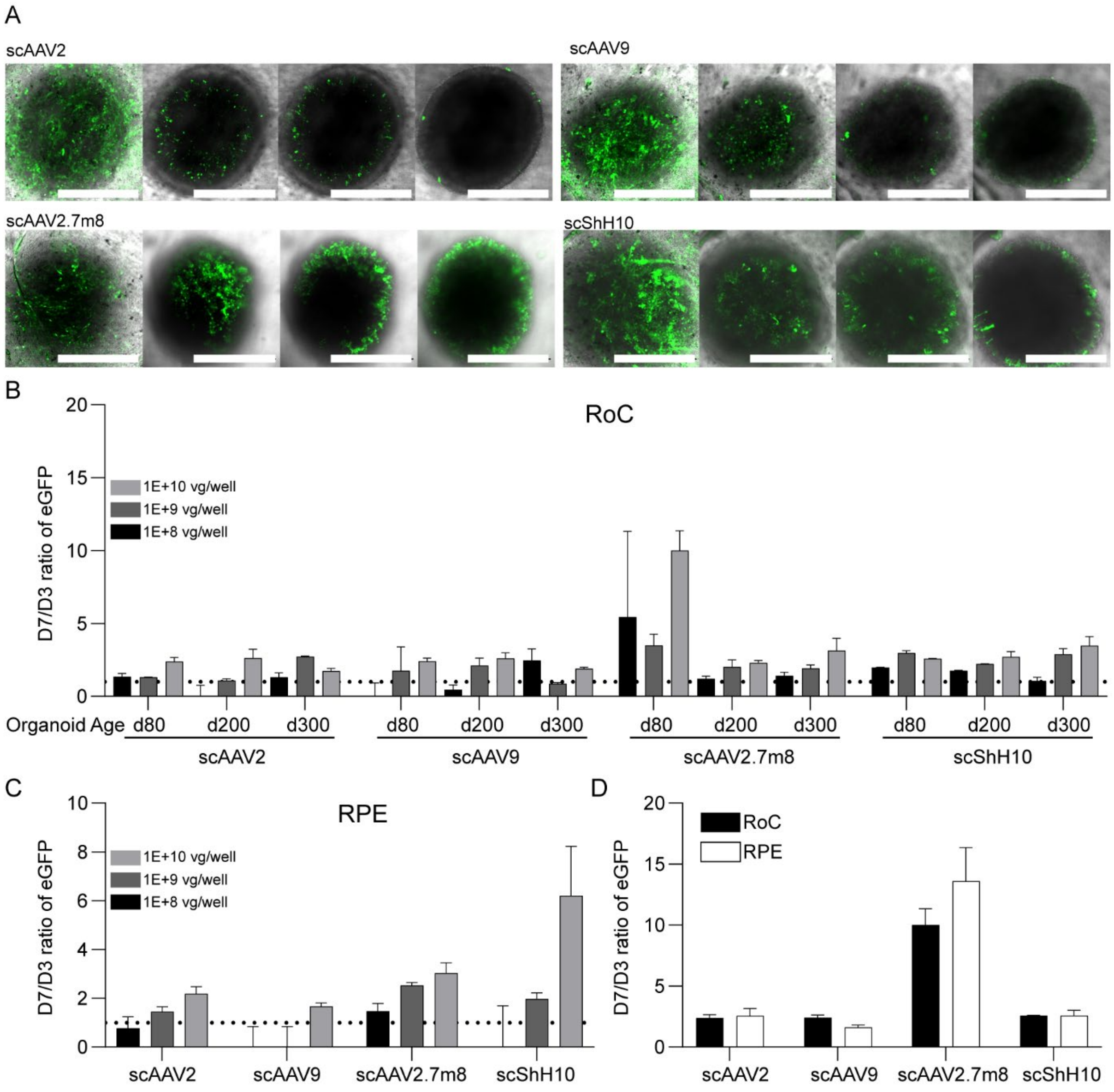

Figure S5. Confocal imaging and kinetics of AAV-transduced RoC. ***Related to Figure 3*****.**
(A) Representative brightfield and confocal eGFP fluorescence live imaging of RoC with day 80 and 300 retinal organoids. The fluorescent images are presented as maximum intensity projections. Scale bars: 500 µm. (B) Relative eGFP signal comparing day 3 and day 7 after transduction with the respective AAV in the RoC discriminated by the retinal organoid age (d80, d200 and d300). Virus load as indicated in the figure legend. n=3 wells from 1 RoC per condition, Mean + SEM. (C) Relative eGFP signal comparing day 3 and day 7 after transduction with the respective AAV in the non-organoid area of the RoC wells. Virus load as indicated in the figure legend. n=3 wells from 1 RoC per condition, Mean + SEM. (D) Relative eGFP signal comparing day 3 and day 7 after transduction with the respective AAV in the RoC and in the non-organoid area of the RoC wells (RPE) treated with the indicated AAV, with a virus load of 1E+10 virus genomes per well. n=3 wells from 1 RoC per condition, Mean + SEM.

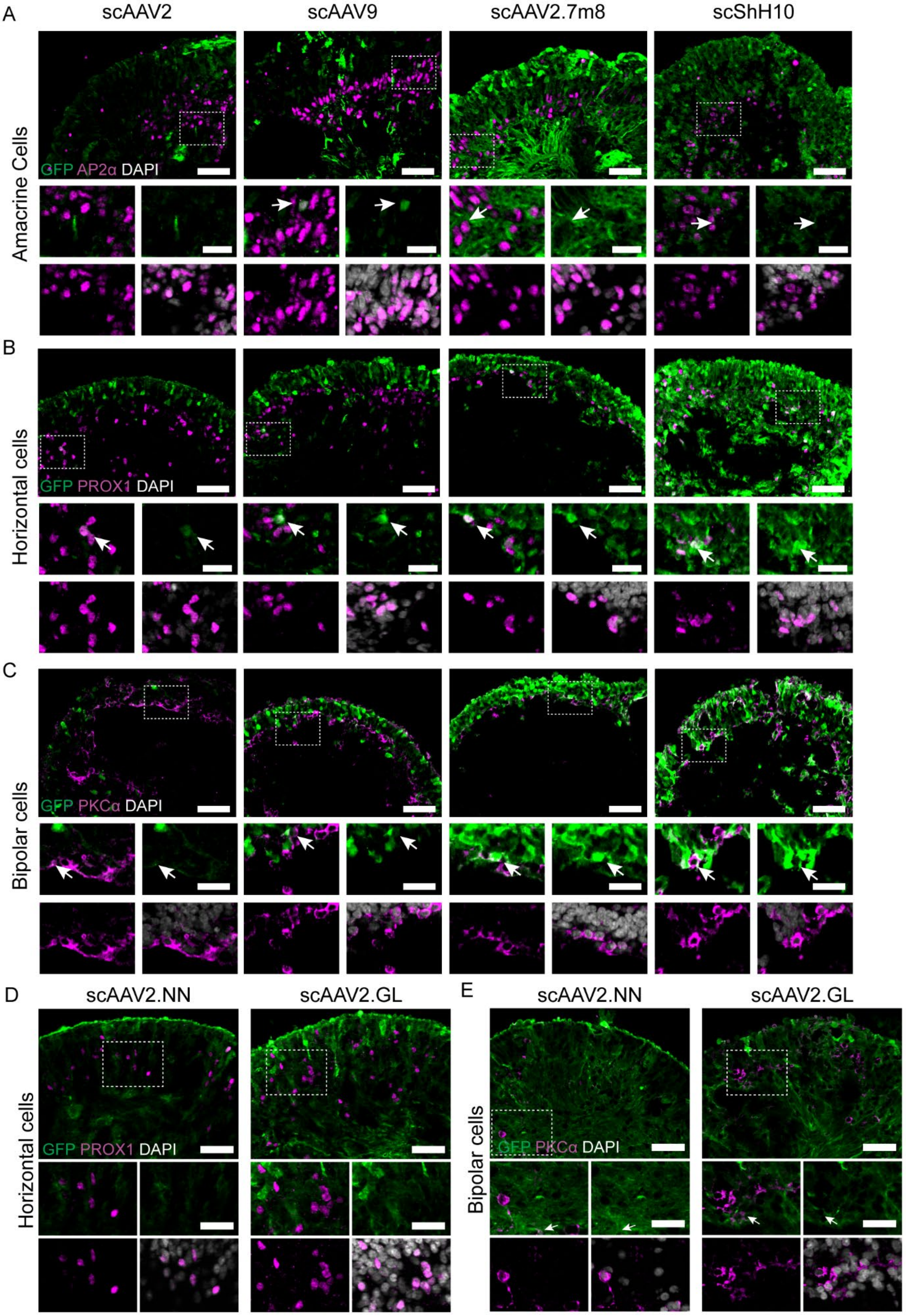
A
scAAV2
scAAV9
scAAV2.7m8
scShH10
Amacrine Cells
GFP AP2α DAPI
B
Horizontal cells
GFP PROX1 DAPI
C
Bipolar cells
GFP PKCα DAPI
D
scAAV2.NN
scAAV2.GL
Horizontal cells
GFP PROX1 DAPI
E
scAAV2.NN
scAAV2.GL
Bipolar cells
GFP PKCα DAPI

Figure S6. Additional data of cell tropism of all tested AAV serotypes in the RoC. ***Related to Figure 4*.**
Retinal organoids in (A) at day 80 were transduced with 1E+10 virus genomes, day 200 (B-C) retinal organoids were transduced with 1E+10 virus genomes. (A-D) Vertical cryo-sections of organoids showing AAV-mediated eGFP expression (green) and cellular co-stainings (magenta) for AP2α (A, Amacrine Cells), PROX1 (B, Horizontal cells) and PKCα (C, Bipolar Cells). Cell nuclei were stained with Hoechst (white). Co-stained cells are highlighted with white arrow. Dotted squares indicate the position of the 4 magnified areas shown below each image. Scale bars: 50 µm (large images), 20 µm (small images). (D-E) Vertical cryo-sections of day 300 retinal organoids transduced with 1E+10 virus genomes showing AAV2.NN and AAV2.GL mediated eGFP expression (green) and cellular co-stainings (magenta) for PROX1 (A, Horizontal Cells) and PKCα (B, Bipolar Cells). Cell nuclei were stained with Hoechst (white). Co-stained cells are highlighted with white arrow. Dotted squares indicate the position of the 4 magnified areas shown below each image. Scale bars: 50 µm (large images), 30 µm (small images).

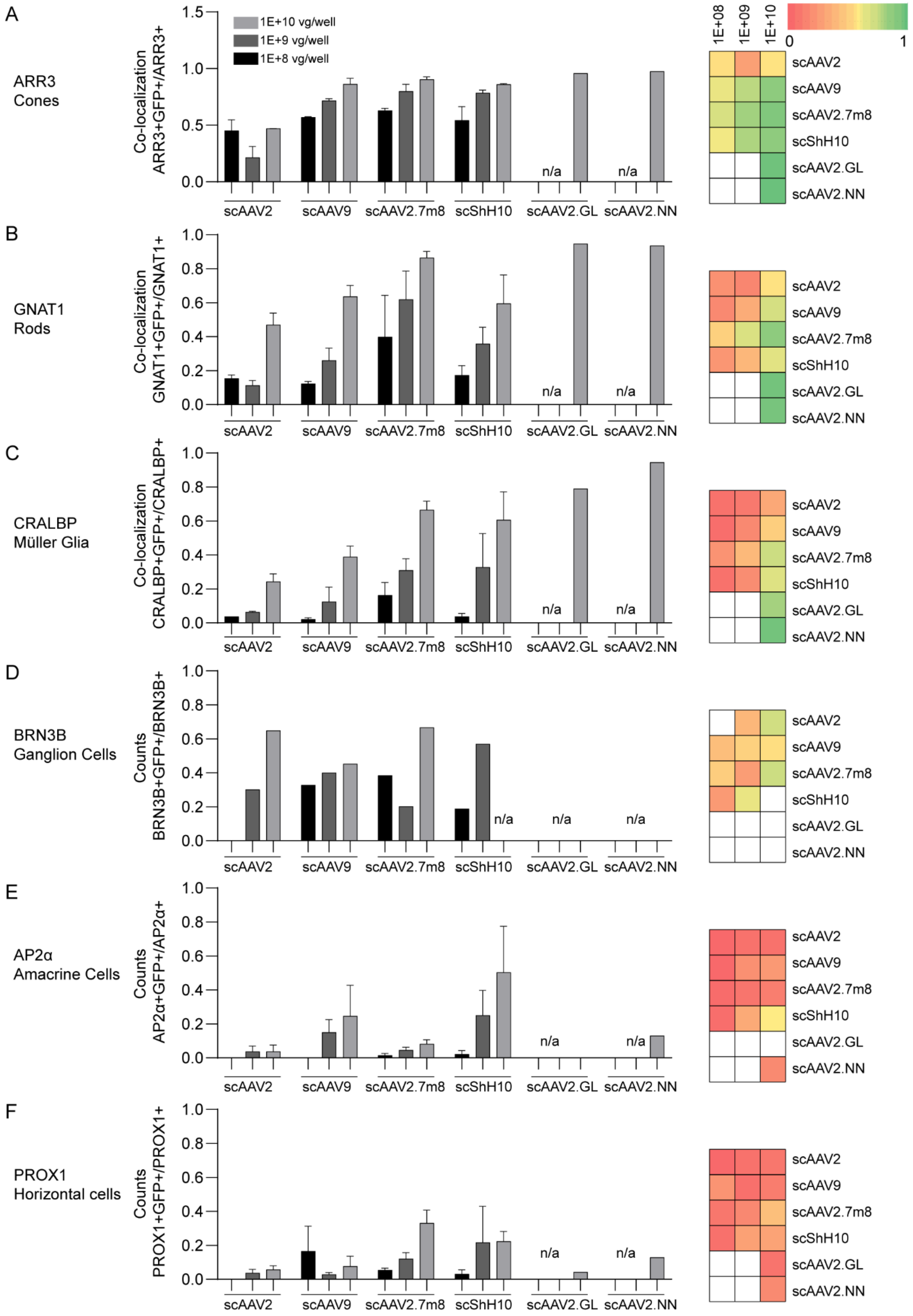

Figure S7. Quantification of AAV cell tropism in the RoC. ***Related to Figure 4 and Figure S6.***
(A-C) Co-localization and (D-F) counting analysis of retinal cell types as shown in Figure 4 and Figure S6. Cell Marker/GFP double positive signals are shown as proportion of the total signal of the individual cell marker's signal. Heatmap on the right shows values of the respective bar graph from 0 (red) to 1 (green) as indicated in

the legend. n/a and respective white squares indicates data points that have not been assessed. (a-c) n=2 RO from 2 RoC (d200 and d300) per condition. (D) n= 1 RO from 1 RoC (d80), (E-F) n=3 RO from 3 RoC (d80, d200 and d300). All graphs are mean + SEM.

Supplemental tables

Table S1. List of primary antibodies and fluorophore-coupled chemicals used

**Antibody**	**Dilution**	**Catalogue number**	**Company**
GFP (whole eye paraffin sections)	1:1500	Ab290	Abcam, UK
GFP (RO sections)	1:1500	GFP-1010	Aves Labs, United States
AP2α	1:100	Sc-12726	Santa Cruz Biotechnology, United States
ARRESTIN 3	1:50	Sc-54355	Santa Cruz Biotechnology, United States
BRN3B	1:50	Sc-31989	Santa Cruz Biotechnology, United States
Cleaved- CASPASE 3	1:200	AP1027	Calbiochem, United States
CRALBP	1:250	Ab15051	Abcam, UK
DESMIN	1:500	M0760	Aligent, United States
Ki-67	1:400	12202	Cell Signaling, United States
PKCα	1:500	Sc-208	Santa Cruz Biotechnology, United States
GNAT1	1:500	GTX114440	GeneTex, United States
Phalloidin Alexa Fluor 647	1:100	A22287	Thermo Fisher Scientific, United States
PROX1	1:2000	ABN278	Merck Millipore, United States
OCT4, SOX2, NANOG, TRA-60, SSEA4 (StemLight Pluripotency Antibodies)	1:200	9656S	Cell Signaling, United States
SOX17	1:500	AF1924	R&D systems, United States
β-III-Tubulin (TUJ)	1:1000	AB0354	Merck Millipore, United States

Table S2. List of genes, sequences of primers and Taqman probes

**Taqman probes**	**Forward**	**Reverse**	**Probe**
eGFP	CTGCTGCCCGACAACCA	TGTGATCGCGCTTCTCGTT	TACCTGAGCACCCAGTCCGCCCT
Murine RNA polymerase II (*Polr2a*)	GCCAAAGACTCCTTCACTCACTGT	TTCCAAGCGGCAAAGAATGT	TGGCTCTTTCAGCATCTCGTGCAGATT
human RNA polymerase II (*RPB1*)	GCAAGCGGATTCCATTTGG	TCTCAGGCCCGTAGTCATCCT	AAGCACCGGACTCTGCCTCACTTCATC
**Commercial primers**	**Company**		
GADPH	Qiagen, Germany		
POU5F1	Qiagen, Germany		
SOX2	Qiagen, Germany		

NANOG	Qiagen, Germany		
SOX17	Qiagen, Germany		
SOX1	Qiagen, Germany		
Pax7	Qiagen, Germany		
**Digital droplet PCR & qPCR probes**			
Primers specific for CMV promotor	CCAAGTACGCCCCCTATTGAC	CTGCCAAGTAGGAAAGTCCCATAAG	
